# What Are the Contemporary Trends on Euphonic Voice Research? A Scientometric Analysis

**DOI:** 10.3390/healthcare10112137

**Published:** 2022-10-27

**Authors:** Clara Puig-Herreros, José Luis Sanz, Vicent Rosell-Clari, Luz Barona, María Melo

**Affiliations:** 1 Department of Basic Psychology, Speech Therapy University Clinic, Universitat de València, 46010 València, Spain; 2 Department of Stomatology, Dental University Clinic, Universitat de València, 46010 València, Spain; 3 Department of Otolaryngology, Barona Clinic, Casa de la Salud Hospital, 46021 València, Spain

**Keywords:** bibliometry, euphonic voice, normal voice, scientometry, speech, voice

## Abstract

(1) Background: The study of the human euphonic voice is a subject that has been researched in recent years from different perspectives. Therefore, it is pertinent to assess the current state of the science. The aim of analyzing the characteristics of normal voice-related publications over the last 11 years is to identify research trends, the numerical and temporal evolution of the publications, their type, and the most-used descriptors. (2) Methods: Bibliometric data from 2011 to 2021 were obtained through several databases. Subsequently, a science mapping analysis was made via VOSviewer software. (3) Results: A total of 901 publications were obtained. The analysis of the scientific production on the field of study regarding the euphonic voice shows a slight increase over the last 11 years, with an average of 82 publications per year. Co-authorship analysis revealed a 6215 authors contributing to the field with a 901 articles (headed by Jiang, J.J. with 18 articles). Keyword co-occurrence analysis highlighted the lack of temporal advancement and variety in the terminology used in the field of voice research. (4) Conclusions: This scientometric study sheds light to the need to broaden in this field of study and the establishment of solid research groups to contribute to its advancement.

## 1. Introduction

Scientometrics is the science which studies the scientific production in order to analyze it from a metric perspective. This type of study not only describes the past of a given topic’s production but also serves to illustrate its evolution over time and detect points of interest in current research via the analysis of the most-used descriptors or keywords [1].

In this sense, through the analysis of objective bibliometric data, scientometric studies help to analyze and characterize scholarly literature by evaluating aspects such as the types of publications, sources, research areas, and institutions, among others [2,3]. Together with the analyses of author collaborations/co-authorship networks and keyword co-occurrence networks (combined use of keywords or descriptors), these parameters can be useful for stakeholders and research groups to identify potential collaborations, tendencies, influential funding agencies, academic institutions, and other useful preliminary data for the planification of future research [4].

In the field of speech diagnosis, pathology, and therapy, the study of the voice is essential. Voice is the sound produced in the larynx by the vibration of the mucosa of the vocal folds as air from the lungs passes through the vocal tract [5,6,7], characterized by acoustic and perceptual parameters such as the pitch, intensity, vocal quality, and resonance [8]. As vocal fold vibration is the main generator of glottal sound, its evaluation is fundamental for the detection, diagnosis, and treatment of vocal pathology [9]. At an objective level, voice presents an almost periodic behavior observable in the glottal or acoustic origin signals through acoustic analysis programs such as spectrograms or cepstrograms [10]. The voice is a crucial instrument of expression and communication (verbal—speaking and singing) [11].

Normality and euphony are the terms used as equivalents to categorize voice as absent of pathology or in a healthy state [12,13]. To the authors’ knowledge, there are no publications that analyze the scientific production on euphonic/normal voice research from a metric perspective. Accordingly, a scientometric research is proposed. The aim of the present study is to analyze scientometric parameters on the scientific production in the field of euphonic/normal voice research in recent years.

The hypothesis of this scientometric study is that scientific production on the field of euphonic/normal voice research has experienced changes in terms of authorship and the studied topics, based on the most-used descriptors, in the studied period. An exponential crescent scientific production is to be expected in this field, showing new areas of interest, while some others remain unexplored.

## 2. Materials and Methods

The present study reports a scientometric analysis on the scientific production related to the normal or euphonic voice in the last 11 years.

### 2.1. Database Search and Study Screening

A general advanced broad search was performed on Web of Science (WoS) electronic database (Clarivate Analytics, London, UK), through its Core Collection (containing information about Sciences, Social Sciences, Arts, and Humanities) and other databases: Current Contents Connect, Data Citation Index, Derwent Innovations Index, KCI-Korean Citation Index, MEDLINE^®^, SciELO Citation Index. A single search in the Web of Science webpage automatically searches in the aforementioned databases.

The search was carried out using the following criteria: Firstly, the search period was limited, as the aim was to provide an updated view on the scientific production related to the normal or euphonic voice. To do this, the level of obsolescence of the articles published in the main journals related to this field was determined, based on the half-life citations. All the journals indexed in the “Audiology & Speech Language Pathology” area from Journal Citation Reports (JCR) repository were selected. The result was 10.33 years, so the search period was set at 11 years, namely from January 2011 to December 2021, both included.

Regarding the search strategy, the sequence was as follows:

[Voice AND (normal OR euphonic) AND (description OR definition OR charact *)]

This search aimed to add knowledge to the current state of the science on the field of study of the definition or characterization of a normal or euphonic voice. No linguistic limits were set. The search was conducted in August 2022.

All results from the broad database search were eligible for the scientometric analysis in order to provide a complete picture of the current state of the available literature related to the database search. Prior to the data extraction and mapping, duplicate records were discarded.

### 2.2. Data Extraction and Mapping

Bibliometric data were obtained from WoS “Analyze Results” tool. The following parameters were extracted: research areas, year of publication, type of publication, language, authors, institution and country of origin, publication and funding sources, terms (keywords, descriptors, or MESH headings).

Additionally, the results from the database search were imported into VOSviewer software tool v1.6.16 (Centre for Science and Technology Studies, Leiden University, Leiden, The Netherlands) for mapping and illustrating bibliometric networks [14]. These bibliometric networks consist of diagrams which exhibit the relationship between the components of a specific metric parameter, i.e., authors or keywords, based on their “relevance”, i.e., number of articles or keyword occurrences, respectively.

Prior to the analysis of the relationships analyzed, a series of limits were established in order to allow for a clear visualization of the general relationships between the metric parameters. Only publications with a maximum of 10 authors and authors with a minimum production of 5 papers were considered to identify the most productive authors. The number of papers produced by each selected author was recorded, as well as the total Link Strength of each one. The Links attribute refers to the number of co-author links from a particular researcher to other researchers. The Total Link Strength attribute refers to the total strength of a particular researcher’s co-authorship links with other researchers based on the number of co-authored papers [14].

Regarding the analysis of the most-used descriptors or keywords, these were obtained from the titles of the studies. In this case, the limit for filtering the minimum number of occurrences of each term was set at 5. For the selection of the descriptors, the VOSviewer software determines the relevance of the term. When creating a map based on text data, general terms, such as “conclusion” and “interesting result”, are generally not used. These terms give very little information, and the usefulness of a map is greater when these types of terms are discarded. To eliminate general terms, the VOSviewer program calculates a relevance score for each term: terms with a high relevance score represent more specific topics related to the data in the text, while terms with a low relevance score tend to of a general nature and are not usually representative of any specific topic. Typically, 40% of terms are excluded based on their relevance score by default. However, unsuitable terms can also be removed manually [15]. Networks were constructed on authors/researchers and descriptors/terms. Items in these networks were linked by co-authorship and co-occurrence, respectively.

To illustrate these networks, the “overlay visualization” setting was used: the size of the label and item is determined by its weight (the higher the weight, the larger the label and item), lines between items represent links between them (co-authorship in the case of authors, co-occurrence in the case of keywords), and the color of the items represent a specific “score”. In the case of “overlay visualization”, colors represent the year of publication, and in the case of “network visualization”, colors represent different research groups.

## 3. Results

The number of publications obtained with the search strategy described above for the period of the last 11 years was 901. Most of them (74.5−82.7% from the total) were extracted from Web of Science Core Collection. From MEDLINE^®^, 524 were obtained (58.2%). From Current Contents Connect, 483 were obtained (53.6%). Other databases yielded the following results: KCI-Korean Journal Database 95 (10.5%), SciELO Citation Index 18 (2%), and Data Citation Index 14 (1.6%).

### 3.1. Temporal Evolution

**Figure 1:**
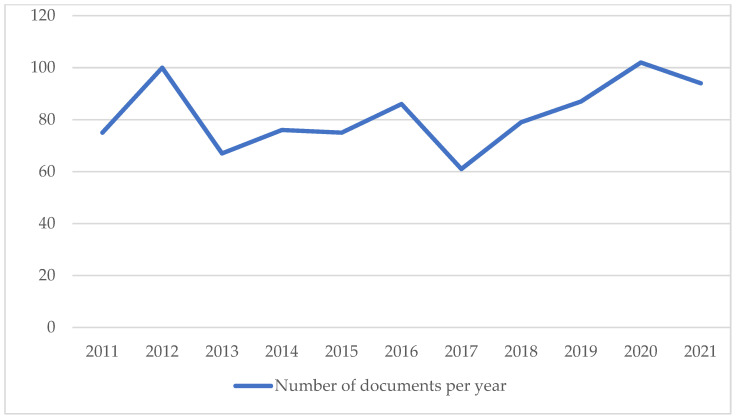
Annual distribution of published articles.

In the time range established for the scientometric analysis, the number of publications that have been produced annually (Figure 1) shows an irregular distribution. In 2011, 75 publications appeared (8.3% of the total in the last 11 years), and in the last year of the study -2021-, 94 appeared (10.4%). The highest point was in 2020, with 102 (11.3%), and the lowest in 2017 (61 -6.7%-).

### 3.2. Type of Publication

Most of the documents are research articles (770 publications: 85.5% of the total). Among them, there are 28 (3.1%) review papers and 28 case reports. Only 14 clinical trials were found (representing only the 1.6% of the total). At the time of the search, 2 early access documents were found, as well as one book. Two editorial materials were also published.

### 3.3. Language

A total of 85% of the publications (766) are in English, but there are also publications in 11 other languages. Among them, the following represent more than 1% of the total publications: Korean (83 publications), Chinese (16), Portuguese, and Spanish (10 each).

### 3.4. Sources

**Figure 2:**
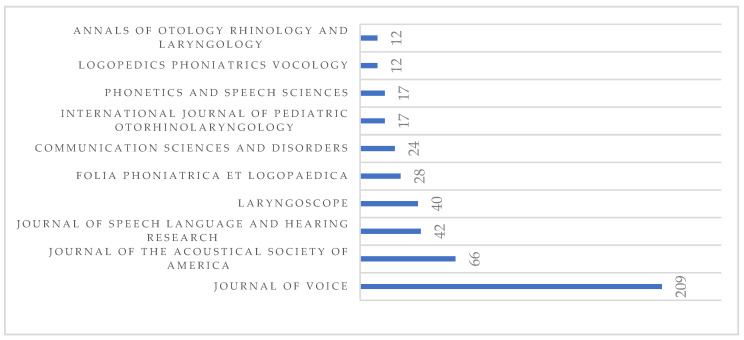
Number of publications per journal (from lowest to highest number).

We found that 524 journals offer articles on “normal voice”. The leading journal in this regard is the Journal of Voice with 209 articles, representing 39.89% of the total number of publications, followed at a distance by other journals with fewer publications; thus, the second journal is the Journal of the Acoustical Society of America with 66 publications (12.6% of the total). Figure 2 shows the 10 journals with the most publications.

### 3.5. Research Areas

The journals showing articles within the field of our search belong to 120 different research areas according to WoS. Among the top 10, based on the total number of publications during the search period” we found “Audiology Speech Language Pathology”, “Psychology”, 4 medical areas (Neurosciences Neurology, Otorhinolaryngology, Respiratory System, and Pediatrics) and 4 from other different areas (Engineering, Computer Science, Behavioral Sciences, and Communication). All research areas belong to the following research domains: Science Technology, Life Sciences Biomedicine, Social Sciences, Technology, Physical Sciences, and Arts and Humanities.

### 3.6. Authors

A total of 6215 different authors contributed to the field of study on the present topic, according to our search strategy. Heading the list are 4 authors: Jiang, J.J., 18 publications; Baskent, D., 16; Choi Seong, H.E.E., 15; and Behlau, M., 14. Following them, with 9 publications: Tayama, N.; and Yamauchi, A., Cielo, C.A., D’Haeseleer, E., Imagawa, H., Sakakibara, K.I., Tayama, N., Xu, W., Yamauchi, A., Yokonishi, H. presented 8 publications each. Finally, with 7 articles: Choi, C.H., D’haeseleer, E., Imagawa, H., Nito, T., Sakakibara, K.-I., and Van Lierde, K.

A total of 78 corporate-authored publications were identified, for which 7 organizations are responsible, including the IEE (Institution of Electrical Engineers) with 58 publications and the International Speech Communications Associations with 13.

The following table shows the “Total Link Strength” and the number of publications for each author. In this case, the data, analyzed with the VOSviewer software tools, are presented. The following filters were applied: only articles written by a maximum of 10 authors and authors appearing in at least five articles were analyzed (Table 1).

**Figure 3:**
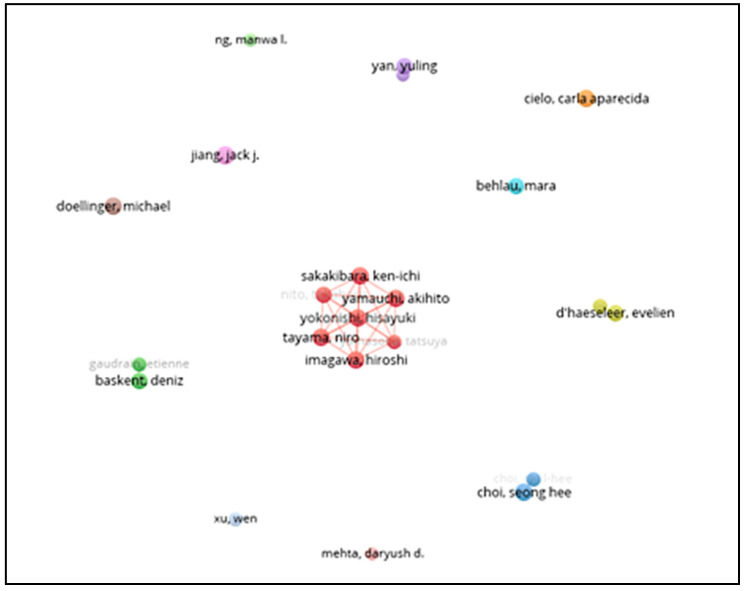
Authors’ network visualization (VOSviewer). Based on data from the most productive authors, established by the aforementioned limits (publications with a maximum of 10 authors and authors with a minimum production of 5 papers).

From the 22 authors identified, not all created co-authorship networks with others. The largest group offers seven elements, and most of them appear individually. The co-authorship networks are shown in Figure 3:

### 3.7. Institutions

**Figure 4:**
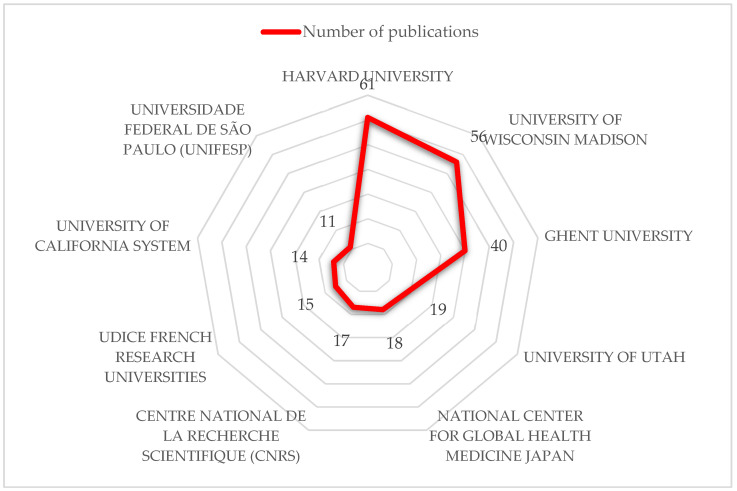
Top 10 institutions with the largest number of publications.

The authors belong to 1730 different academic, health, or research institutions. Figure 4 shows the number of publications produced by the top 10 institutions (including Harvard University with 61 publications and Universidade Federal de São Paulo with 11).

### 3.8. Countries

**Figure 5:**
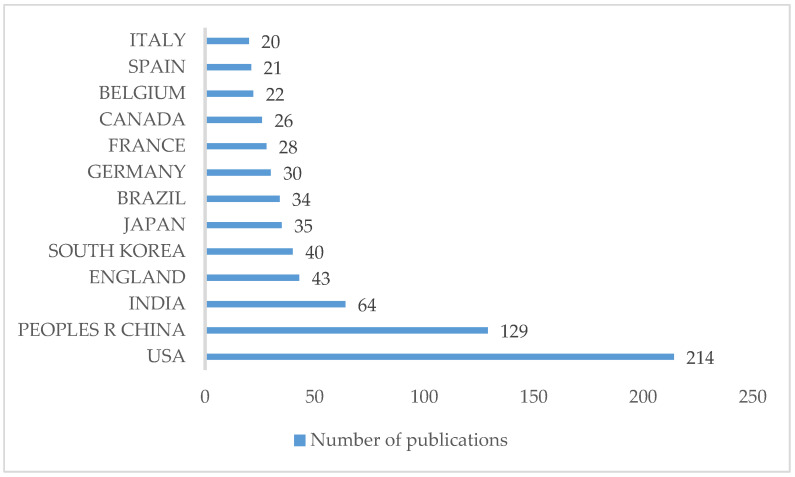
Countries with the highest number of publications.

Scientific articles were produced by institutions located in 73 different countries; 13 countries have 20 or more articles (Figure 5), led by the USA with 214 publications, accounting for more than three quarters (78.2%).

### 3.9. Funding

From the publications, 60.4% (502) did not present external funding. In the cases in which it is reported, it is mostly from governmental institutions (generally national, but also international, such as the European Commission).

### 3.10. MESH Headings

We found that 51.2% of the publications do not use MESH terms. From these, 962 were detected. The 25 most frequently used MESH terms are listed below with an indication of the number of times they were found and their percentage within the 901 articles included in our study (Table 2). Among them, the most-used term was “humans” with 450 occurrences (49.9% of the total), and the least used term was “aged 80 and over” with 50 occurrences (5.5%).

### 3.11. Terms

**Figure 6:**
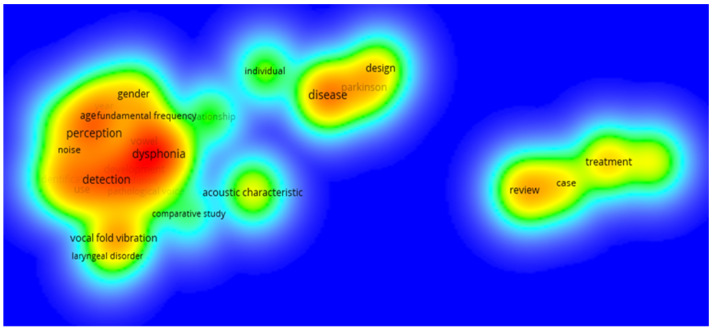
Most-used keywords (the redder color indicates a more frequent use, and the green color less frequent use). Based on data from the most common keywords, established by the aforementioned limits (the number of occurrences of each term was set at 5).

A total of 2643 different keywords appeared in the study sample. In order to visualize the most frequently used keywords or expressions, a minimum number of appearances—five—was established, reducing the number of keywords to 69. Then, the bibliometric software tool was used to delete the less common descriptors (28 of them), as previously described. Finally, three other general terms (“person”, “patient”, and “research”) identified by the system with a very low level of relevance were deleted, so 38 terms were considered (Table 3):

The terms “detection” (23), “dysphonia” (19), “perception” (18), “disease” (18) and “treatment” (16) were the five most-used terms found on the study period. Figure 6 illustrates the analyzed keywords by clusters.

Figure 6 shows that the most-used term is “dysphonia”, which is surrounded by others such as “gender”, “age” (demographic factors), “detection”, “perception”, “noise”, “fundamental frequency” (perceptual/acoustic aspects), and “vocal fold vibration” and “laryngeal disorder” (anatomophysiological factors). Very close but with less relevance, the term “acoustic characteristic” appears. A little further away and with less occurrence in the search is the cluster that includes “disease”, “design”, “individual”, and “Parkinson”, which also indicates the importance that has been given to the study of the voice with regards with this disease in recent years. The most distant cluster from the vocal aspect itself is composed of the terms “treatment”, “case”, and “review”, which may indicate the importance of studying what exists in the current literature on a particular topic through reviews, in addition to knowing the treatment of some vocal pathologies via case studies.

**Figure 7:**
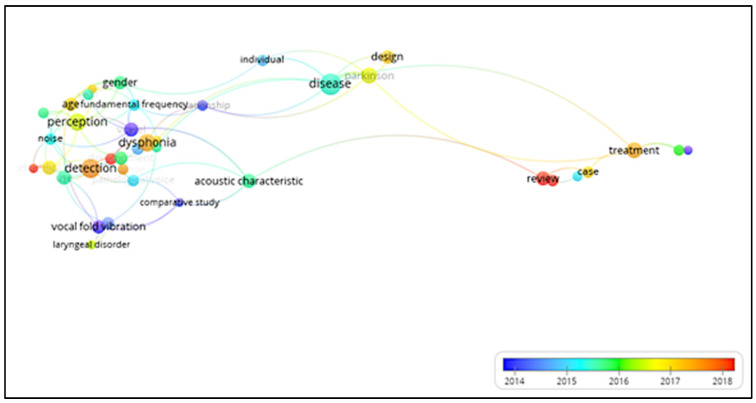
Keyword co-occurrence showing changes over the studied period. Blue colors identify the most-used terms in the initial part of the period.

Figure 7 illustrated the above-mentioned keywords/descriptors and the change in their use over time. It can be observed that the most-used term from 2018 until now is “review”. Although the terms “dysphonia” and “detection” had higher relevance previously (2017–2018), they are also considered relevant terms, as well as “disease” (2015–2016) by the size of the circle.

## 4. Discussion

The present study on scientific production aimed to investigate the scientific production on the normal/euphonic voice from a metric perspective. With regards to voice, the term normality refers to the voice of an individual which has a pleasant vocal timbre, a tone appropriate to sex and age, and a volume appropriate to their vocal needs [13]. Parallelly, euphony is defined as the sound of good quality for listeners and the sound produced without difficulty or discomfort for speakers in a sustained manner [14]. Nonetheless, these terms are often used indistinctively as synonyms. For this reason, both terms were used as descriptors in the search strategy to ensure that no article on the field of nonpathological voice research was excluded.

Considering the large scientific production in any field of science, we set limits to the period of study, as performed in previous studies: previous bibliometric studies offer very different limits, from 67 years [15] to 1 year [16], or even 9 years in Calvache’s study [17]. To use an objective criterion, the average cited half-life was calculated for the 27 journals specialized in the field of Audiology and Speech Language Pathology in Journal Citation Reports (JCR). This measure ranges from 3 years (Trends in Hearing) to 25.2 years (Phonetica). The number of publications included in the study was 901 for a period of 11 years. The work by Pestana [16] includes 162 for a period six times longer, although focused on a very specific aspect, the sung voice.

Previous bibliometric studies have focused on specific aspects, such as the sung voice [15], the analysis of sex bias in research and publications in the area of laryngology [16], scientific production on physiological vocal rehabilitation [18], phono-audiological interventions in spasmodic dysphonia [17], the relationship between vocal production, bilingualism [19], etc.

Our analysis has been performed on scientific articles. This is the main source of what in bibliometrics is known as primary documents, i.e., those containing the most immediately presented research (together with congress presentations). This type of document is collected in repositories and databases that allow its classification, management, measurement, and search. For this study, seven databases were used for the bibliometric analysis, WoS Core Collection being the main source of information. These databases were selected due to their available bibliometric tools and the possibility to export data to other software.

However, databases have some limitations. A common one is the lack of information due to defects in the published article or in the register and collection of data in a repository. Nevertheless, this problem does not occur with some data, such as the year of publication, language, and type of document. Others present some errors. For example, 1% of the publications (9 of 902) have no authors. This can be partially explained by the presence of letters (0.2%), editorial comments (0.2%), and other similar material. We also found that 2.7% of the publications are not classified by research area, and three publications (0.3%) have no registered source (journal, book…). The absence of the authors’ affiliation to an institution is also common. In addition, MESH terms are not used in almost half of the publications (48.8%). Finally, the most common lack of information is that related to funding resources. This information is missing in 48.8% of the cases. Probably, although they are not cited, much research is funded by universities, since they are the institutions that offer more scientific production.

Another question is the possible repetition of data. In fact, it is common to find the same author with different identification. For instance, changing names and surnames or the case of double name/surname. This situation can be found with the names of institutions as well. In the present study, we have revised this kind of diverse data before presenting the data analysis.

One of the general rules of scientometrics is that scientific production grows exponentially [20]. Interestingly, this was not the case in the present study. The annual evolution of the number of research articles published does not follow this type of growth, but rather, over the last 11 years, it shows an irregular pattern with ups and downs. Nevertheless, a slight increase of 19 publications can be observed. This suggests a stability that could be interpreted as a lack of research in this field, which should be more extensive, for example, in terms of epidemiological studies. In this sense, the absence of a term such as “epidemiology” or “index” and the low relevance of others such as “prevalence” is evident (Table 3).

The bibliometric postulate that a small number of authors generate most publications [21] is not fulfilled in this case either. In the present study, 6215 different authors were detected, but a limited number of publications were found from each of them. For example, the most productive author (Jiang, J.J.) contributed with 18 publications, representing 2% of the total.

On the contrary, the scientific production analyzed complies with the principle of dispersion [22], showing that 901 articles are distributed among 633 journals, distributed in 120 different research areas, including those not related with health sciences.

Keywords or descriptors are a fundamental part of scientific documents, as they allow them to be indexed and classified in databases to enable thematic searches. Knowing the most-used keywords can help to guide and refine future searches and systematic reviews of the scientific literature. Co-occurrence analysis is useful in this regard [17,18,19,20,21,22,23]. It is evident that authors do not always use standardized terms (such as MESH or EMTREE), mainly due to the limitations of the list and the continuous advance of research in new fields of knowledge.

VOSviewer software is a useful bibliometric tool, as it is able detect and present data and their relationships. In addition, it allows one to individualize the research, as some limits or characteristics of the bibliometric study can be made by the researcher, even selecting terms (among those offered by the software), as described in the text. In this case, limits were placed on the authors and keywords, as performed by other scientometric analyses in the field of medical sciences [24,25]. This was performed to allow for a clear visualization of the general relationships between the metric components. However, it can be argued that this poses a limitation, since both less productive authors and less common or relevant terms are discarded in these analyses.

The “overlay” view presented in Figure 7 shows a range of colors indicating the temporality of the appearance of the terms. Most of the keywords are colored in blue, which is a further indication of the information provided by the analysis of the temporal evolution: the same topics are still being researched in recent years. However, new contributions appear, such as “dysphonia”, “detection”, “review”, “case report”, “treatment”, “COVID”, “validation”, “prevalence”, “age”, and “formant frequency”, but with different intensity of expression.

Within the limitations of our analysis, a predominance of keywords related to pathology and its treatment can be observed as well as a deficit in terms of diagnosis-related keywords. Nevertheless, prior to the studied period addressed in this study, a series of publications on voice diagnosis were developed, mainly related to acoustic characteristics [26,27,28].

Thus, regarding the initial hypothesis, we can accept that there have been advancements in terms of new areas of interest, while others are less considered. However, we must reject the hypothesis partially in relation to the scientific production growth because, even though a slight increase is detected, it does not follow an exponential increase.

Altogether, the results from this study can facilitate future research in the field and provide readers with an updated view of the past and the present of the scientific production in the field of normal/euphonic voice research and the varying interest of the different topics. The presented data can be of use as a starting point from which to fill existing knowledge gaps within this subject, based on the most-used keywords. Additionally, researchers in the field can benefit from data such as suitable journals, most productive authors, or sources of funding for future research.

## 5. Conclusions

Under the conditions of this study, we can conclude that the analysis of the scientific production on the field of study regarding the euphonic voice shows a slight increase over the last 11 years (75 in 2011 vs. 102 in 2021), with an average of 82 publications per year. Studies in this area are distributed among different specialized and general journals. Keyword co-occurrence analysis highlighted the lack of variety in the terminology used in the field of voice and the lack of increase in variety over the studied period. This scientometric study sheds light to the need for diversification in this field of study and the establishment of solid research groups to contribute to its advancement. More research is needed in the field of the characterization of the euphonic voice and the related conditions and involved factors, such as epidemiological studies in different populations.

## Figures and Tables

**Table 1:** Authors in order of highest to lowest Total Link Strength.

Author	Total Link Strength	Number of Documents
Imagawa, H.	45	8
Sakakibara, K.-I.	45	8
Tayama, N.	45	8
Yamauchi, A.	45	8
Yokonishi, H.	45	8
Nito, T.	41	7
Yamasoba, T.	36	6
Choi, C.-H.	6	6
Choi, S.H.	6	8
D’Haeseleer, E.	6	7
Van Lierde, K.	6	6
Baskent, D.	5	7
Gaudrian, E.	5	6
Murry, T.	1	5
Yan, Y.	1	7
Behlau, M.	0	7
Cielo, C.A.	0	8
Doellinger, M.	0	8
Jiang, J.J.	0	9
Metha, D.D.	0	5
Ng, M.L.	0	5
Xu, W.	0	6

**Table 2:** Most frequently used MESH terms.

MESH Terms	Umber of Occurrences (%)
Humans	450 (49.9)
Female	345 (38.3)
Male	328 (36.4)
Adult	236 (26.2)
Voice Quality	190 (21.1)
Middle Aged	171 (19.0)
Speech Acoustics	160 (17.8)
Young Adult	153 (17.0)
Aged	118 (13.1)
Adolescent	096 (10.7)
Speech Production Measurement	095 (10.5)
Phonation	090 (10.0)
Child	088 (09.8)
Vocal Cords	080 (08.9)
Voice	080 (08.9)
Acoustics	079 (08.8)
Voice Disorders	079 (08.8)
Speech Perception	075 (08.3)
Case Control Studies	062 (06.9)
Dysphonia	059 (06.5)
Time Factors	057 (06.3)
Speech	054 (06.0)
Sound Spectrography	053 (05.9)
Phonetics	051 (05.7)
Aged 80 And Over	050 (05.5)

**Table 3:** Selected terms, level of relevance assigned by the VOSviewer software, and number of occurrences.

Term	Occurrence
Detection	23
Dysphonia	19
Perception	18
Disease	18
Treatment	16
Parkinson	16
Review	13
Use	13
Vowel	13
Acoustic characteristic	12
Identification	12
Vocal fold vibration	11
Development	11
Age	11
Gender	11
Design	11
Impact	10
High speed digital imaging	10
Fundamental frequency	9
Case	8
COVID	8
Case report	8
Pathological voice	8
Noise	8
Individual	8
Adductor spasmodic dysphonia	7
Validation	7
Year	7
Relationship	7
Speech intelligibility	7
Vocal fold	6
Role	6
Influence	5
Larygeal disorder	5
Older adult	5
Comparative study	5
Formant frequency	5
Prevalence	5

## Data Availability

The data presented in this study are available on request from the corresponding author.
